# Sexual Assault Among Young Adolescents in Informal Settlements in Nairobi, Kenya: Findings from the IMPower and SOS Cluster-Randomized Controlled Trial

**DOI:** 10.1007/s11121-023-01595-1

**Published:** 2023-11-15

**Authors:** Clea Sarnquist, Rina Friedberg, Evan T. R. Rosenman, Mary Amuyunzu-Nyamongo, Gavin Nyairo, Michael Baiocchi

**Affiliations:** 1 Department of Pediatrics, Stanford University School of Medicine, 300 Pasteur Drive, Stanford, CA 94305-5208 USA; 2 LinkedIn Data Science and Applied Research, 1000 West Maude Ave, Sunnyvale, CA 94085 USA; 3 Department of Mathematical Sciences, Claremont McKenna College, 850 Columbia Avenue, Claremont, CA 91711 USA; 4 Africa Institute for Health and Development, 7th Floor Suite B, Wood Avenue/Kindaruma Road Junction, Box 45259, Nairobi, Kenya USA; 5 Department of Epidemiology and Population Health, Stanford University, 150 Governor’s Lane, Stanford, CA 94305-5405 USA

**Keywords:** Sexual assault, Young adolescents, Informal settlements, Schools, Global health, Empowerment self-defense

## Abstract

Sexual assault is a global threat to adolescent health, but empowerment self-defense (ESD) interventions have shown promise for prevention. This study evaluated the joint implementation of a girls’ ESD program and a concurrent boys’ program, implemented via a cluster-randomized controlled trial in informal settlements of Nairobi, Kenya, from January 2016 to October 2018. Schools were randomized to the 12-h intervention or 2-h standard of care. Students were randomly sampled to complete surveys at baseline and again at 24 months post-intervention. A total of 3263 girls, ages 10–14, who completed both baseline and follow-up surveys were analyzed; weights were adjusted for dropout. At follow-up, 5.9% (*n* = 194/3263) of girls reported having been raped in the prior 12 months. Odds of reporting rape were not significantly different in the intervention versus SOC group (OR: 1.21; 95% CI (0.40, 5.21), *p* = 0.63). Secondary outcomes, social self-efficacy (OR: 1.08; 95% CI (0.95, 1.22), *p* = 0.22), emotional self-efficacy (OR 1.07; 95% CI (0.89, 1.29), *p* = 0.49), and academic self-efficacy (OR: 0.90; 95% CI (0.82, 1.00), *p* = 0.06) were not significantly different. Exploratory analyses of boys’ victimization and perpetration are reported. This study improved on previous ESD studies in this setting with longitudinal follow-up of individuals and independent data collection. This study did not show an effect of the intervention on self-reported rape; findings should be interpreted cautiously due to limitations. Sexual assault rates are high in this young population, underscoring a dire need to implement and rigorously test sexual assault prevention interventions in this setting. The trial was registered with Clinical Trials.gov # NCT02771132. Version 3.1 registered on May 2017, first participant enrolled January 2017.

**Supplementary Information:**

The online version contains supplementary material available at 10.1007/s11121-023-01595-1.

## Introduction

Experiencing sexual assault during the key developmental period of adolescence can have serious and lifelong consequences, ranging from mental and physical health challenges to early childbearing and school dropout, all of which limit potential (Guerra et al., 2018; Houtepen et al., 2020). Prior victimization is also a risk factor for future victimization (Gidycz et al., 1993). Thus, it is essential to implement prevention programs early (Assink et al., 2019; United Nations Children’s Fund, 2014).

Although young adolescents are at high risk of sexual assault, most previous research has focused on older populations (14–25 years of age) and high-income countries. Nonetheless, literature on adolescents living in the informal settlements of Nairobi, Kenya, shows high rates of rape, between 8 and 25% annually (Baiocchi et al., 2017; Sarnquist et al., 2014a, b), depending on age.

In a study reporting on baseline data from this randomized trial, Baiocchi et al. (2019) found that the study population is particularly vulnerable, with only 82.9% of girls reporting having both parents and 1% reporting having no parent. At baseline, 49.9% reported it would be difficult or very difficult to get 1000 Kenyan shillings for an emergency. Demonstrating a significant need for an intervention, at baseline, 6.5% of the girls in the study reported having been raped in the prior 12 months. The study further found, for girls in this study, the majority (65.7%) of perpetrators of rape were a boyfriend (typically an older male), followed by strangers (17.4%) and relatives (13.1%).

Empowerment self-defense (ESD) interventions, such as the one studied here, have not previously been evaluated in a low-income country with as rigorous a study design. In Nairobi, prior research showed a significantly decreased incidence of sexual assault among secondary-school and upper primary-aged adolescents with similar interventions (Baiocchi et al., 2017; Sarnquist et al., 2014a, b; Sinclair et al., 2013). However, this research had several design limitations: (i) no randomization, (ii) no longitudinal follow-up of individuals (yielding an inability to account for study dropout), and (iii) no independent data collection (i.e., the same organization deployed the intervention and collected outcome data). A study of related interventions, with the same limitations, in primary and secondary schools in Malawi, reported significant decreases in sexual violence (risk ratio [RR] 0.68, 95% CI (0.56, 0.82)) (Decker et al., 2018). In Canada, the USA, and New Zealand, ESD programs, primarily among college-age women and with shorter follow-up periods, have shown significant reductions in sexual assault (Hollander, 2014; Jordan & Mossman, 2018; Senn et al., 2015).

The current trial assessed the effectiveness of a novel combination of an existing girls’ empowerment self-defense intervention (IMPower) and a new intervention for boys, designed to engage boys for a similar time period and complement the girls’ intervention (Sources of Strength, or SOS). The hypothesis was that the combined intervention would reduce the rate of rape of girls in treatment schools compared to control schools. This study improves upon the scientific rigor of the studies in Nairobi and Malawi by incorporating longitudinal follow-up of participants and using an independent data collection organization.

### Intervention

As described in this study’s protocol paper, the IMPower and SOS interventions were developed by No Means No Worldwide (NMNW) to target younger adolescents in Nairobi (Sarnquist et al., 2019). Both the girls’ and boys’ interventions were taught in six 2-h sessions held weekly on school property. Sessions in the intervention included role plays, facilitated discussions, and verbal and physical skills practice.

The girls’ intervention session topics included: (1) building rapport and self-esteem and providing definitions and objectives; (2) personal awareness, self-efficacy, boundaries, and assertive communication skills; (3) verbal and physical defense skills; (4) review of verbal and physical skills and skills practice using bags and mitts; (5) de-escalation and negotiation, and more advanced defense techniques for use in instances such as multiple or armed attackers; and finally (6) review of all previous sessions and a discussion of sexual assault and harassment experiences. The IMpower program is informed by the Acknowledge, Assess, and Act (AAA) theory (Rozee & Koss, 2001), which is built upon a prior cognitive ecological model of women’s resistance to sexual coercion (Nurius & Norris, 1996).

The boys’ intervention topics included (1) building rapport and developing awareness about gender interactions; (2) personal awareness and learning assertive body language and verbal response; (3) intervention and verbal and physical defense skills; (4) defining and understanding sexual consent, valid consent, causes and myths about rape, and de-escalation and negotiation techniques; (5) responsibility for one’s actions and behaviors and practice using intervention skills; and finally (6) reviewing key concepts, reinforcing skills through role plays, and public commitments to utilize new skills.

### Standard of Care

The standard of care (SOC) group received a one-time 1.5 to 2-h life skills class modelled on a curriculum required by the Kenyan Ministry of Education. The SOC sessions were also taught by trained NMNW facilitators on school property. The curriculum included a variety of topics, including sexual assault, sanitation, food safety, and personal rights. Though sexual assault is discussed in the SOC, there was no overlap in the skills transferred in the SOC and the intervention.

After the completion of the study and post-final data collection, a sample of schools in the SOC arm were offered the intervention, led by NMNW-trained facilitators.

## Methods

### Study Design and Population

This is the primary analysis for a previously described cluster-randomized controlled trial (CRT) (Sarnquist et al., 2019). Data were collected January 2016–October 2018 at co-educational schools in five informal settlements of Nairobi, Kenya. The settlements are characterized by severe poverty, lack of infrastructure such as lighting and secure housing materials, and high rates of violence generally. Previous work in these five settlements recorded a self-reported annual incidence of sexual assault of 17.9% for high school women, and a self-reported annual incidence of rape of 7.3% for middle school girls (Baiocchi et al., 2017; Sarnquist et al., 2014b).

**Fig. 1:**
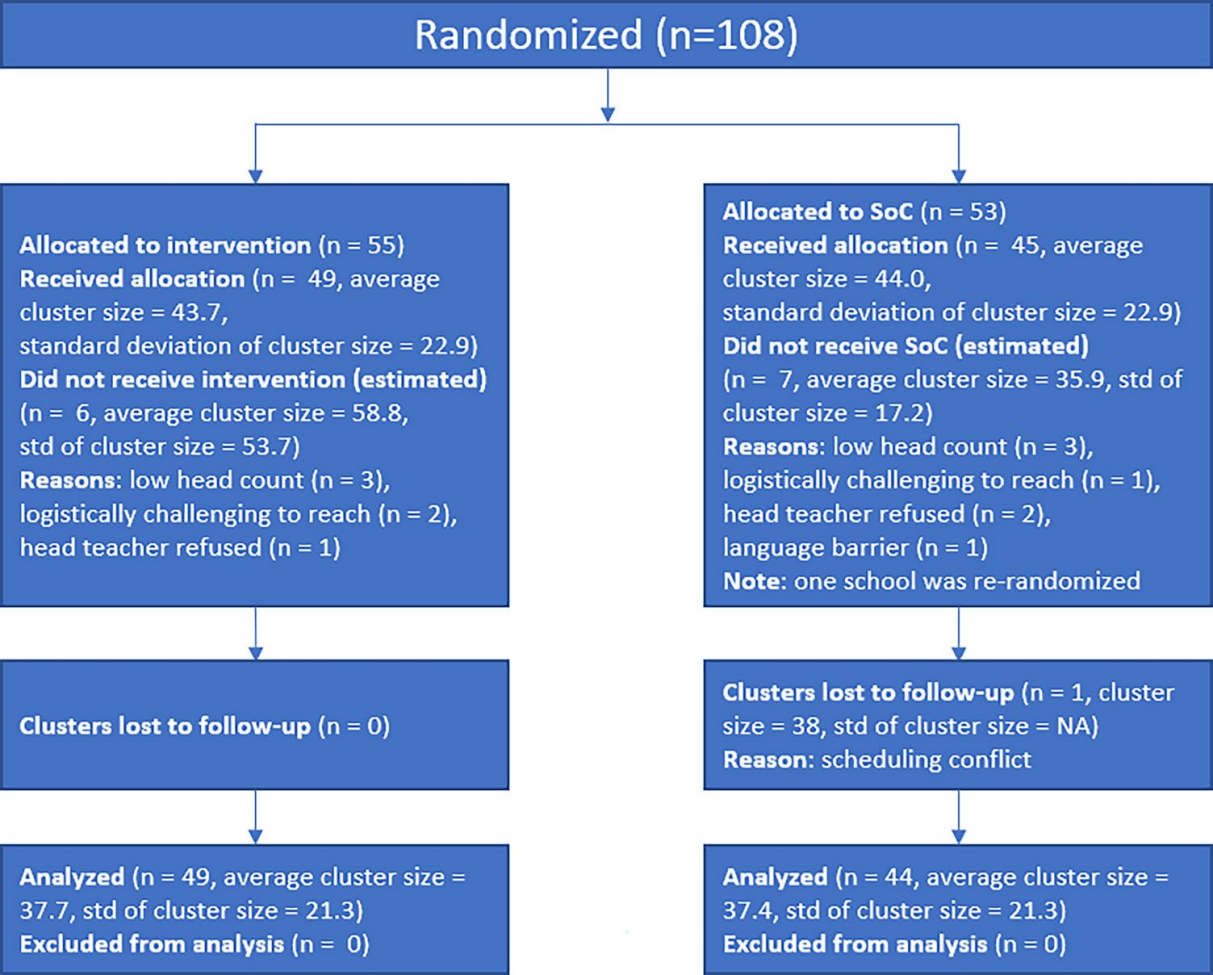
While 108 schools were randomized, 107 unique schools were allocated (one school was randomized twice and was assigned the same assignment both times)

The primary inclusion criterion was being a primary school that included classes 5–8 (approximately 10–14 years old) that had not had the intervention in the previous 3 years, as different forms of this intervention had been taught in some of these settlements for 10 years. Trainers from NMNW recruited schools for participation. A total of 107 unique schools were matched and randomized across the five settlements prior to recruitment. Of the 107, 94 (88%) participated and 13 (12%) did not (Fig. 1).

Of the 13 that did not participate, 10 were removed by the study team before initiation of data collection for a low number of students (6 schools), logistical challenges (3 schools), or language barriers (1 school). Three schools had head teachers who declined to participate before the beginning of data collection. Within the matched pairs that agreed to participate, an independent Bernoulli trial with a 50% probability was used to allocate one school to the intervention group and the other to the standard of care (SOC) group. The study statistician generated the allocation sequence at the cluster level (school level). Students were recruited and enrolled in the study after the randomization of the schools. Participants were randomly sampled by drawing colored beads from an opaque bag. Students who drew beads of a pre-determined color, up to 101 girls per school, were invited to participate in the surveys.

While the main study was intended to be an evaluation of the intervention’s impact on girls, we included data collection on boys. Given resource constraints and the early-stage development of the SOS program, the study was not designed to evaluate the boys’ intervention to the same standard as the girls’ and should therefore be considered a “pilot” study. The boys’ sub-study had the same methods with a few notable differences: (i) one intervention arm school only taught girls, so was excluded from the boys’ study, (ii) the sample sizes were typically 10–20 boys per school, and (iii) the surveys included questions about perpetration of sexual assault.

### Ethics and Safety

The intervention was developed by the non-governmental organization (NGO) No Means No Worldwide (NMNW) and implemented by a different NGO, Ujamaa Africa. Trainers were chosen through an intensive interview and vetting process to ensure that they were respected members of their communities and had a background in health improvement. All trainers received extensive instruction from expert facilitators and participated in field training exercises conducted outside of the study area. Trainers were required to pass written and oral examinations, as well as a physical skills demonstration before teaching the curriculum. The training and field exercises combined took approximately 1 year to complete before trainers were allowed to teach classes alone.

Data were collected by the independent, Nairobi-based research firm African Institute for Health and Development (AIHD). Data collection teams were trained annually in the study’s specific survey instrument and population; these trainings were all co-led by AIHD and Stanford University, with Ujamaa staff invited to participate. Survey data were collected on paper as the data collection teams determined that it was too risky to take electronics into these informal settlements; safety took precedence over improvements in data collection on sexual and risk behavior data with electronic collection (Gnambs & Kaspar, 2015). Survey responses were confidential, but those participants who reported sexual assault to research team members were referred to the Sexual Assault Survivors Anonymous (SASA) support groups run by Ujamaa and NMNW, as well as to medical services, as needed. Additional referral resources include the Center for Rights Education and Awareness (which provides integrated services for survivors and legal support), the Gender Violence Recovery Centre at Nairobi Women’s Hospital, Medecins Sans Frontieres’ Centre for Victims of Sexual Violence in Kibera, and Childline Kenya (a national toll-free telephone and web-based helpline for children). Ethics and informed consent data can be found in the Declarations following this manuscript. Overall, 13 of the 14 recently published (2023) recommendations for ethical research on violence against women and children were followed (Peterman et al., 2023). The sole exception was mandated reporting of violence against children, which is complicated in a setting where such mandates do not exist and reporting may endanger children further. Participant feedback on the intervention is not described here but was collected via previously published qualitative interviews (Mphamba et al., 2022).

### Outcomes

The primary outcome was girls’ self-report of rape, defined as having been forced into penetrative sex by physical force, threats and intimidation, or coercion, and including both intimate partner violence (IPV) and non-partner violence (NPV). See the Supplementary Information (SI) for question wording. Secondary outcomes included academic, social, and emotional self-efficacy; at the time of this study’s deployment, no validated measures of empowerment in this age group existed so these were used as approximate measures. In a previous randomized trial (Baiocchi et al., 2017) in these settlements, a measure of generalized self-efficacy was found to be increased by IMPower. In addition, several mental health measures, as described elsewhere, were added to the follow-up survey as the result of additional independent funding (Friedberg et al., 2023). An exploratory aim was to investigate how the intervention affected boys; this was both to provide a quantitative foundation for future studies as well as an understanding of how this novel intervention is linked to perpetration and victimization.

### Measures

Scales previously validated or widely utilized in similar populations were used when available. These included (i) questions from the Kenya Violence Against Children Survey (VACS Kenya, 2012), (ii) a slightly revised and shortened version of the World Health Organization’s violence questions (Violence Against Women Prevalence Estimates, 2021), (iii) the “Self-Efficacy Questionnaire for Children” (Ozer & Bandura, 1990), and (iv) domains such as regionally appropriate socioeconomic measures from surveys used to evaluate Stepping Stones (Jewkes et al., 2014), a gender-based violence prevention intervention. Few validated measures for this age group exist, but both the VACS questions and the self-efficacy questions have been tested in this age group. All survey items were reviewed by Kenyan researchers, piloted with members of the target group, and adapted as necessary. For more details about metric selection and adaptation, see this study’s protocol paper (Sarnquist et al., 2019).

Socioeconomic status was measured by the question “If a person became ill in your home and 1000 shillings were needed for hospital or medicines, would you say it would be very easy, easy, quite difficult, or very difficult to find the money?” This question was used due to difficulty in assessing socioeconomic status through traditional measures, such as household income, given the young age of participants. Existing data suggest virtually all households in the informal settlements in Nairobi live on incomes below the World Bank-defined poverty line of $1.90 per day for a family of four.

### Data Analysis

The primary outcome was analyzed with a generalized linear mixed model (GLMM). 95% confidence intervals were computed by a clustered bootstrap with 1000 replicates, and *p*-values were computed via clustered permutation tests (Efron, 1979). This model estimated the log odds ratio (OR) of rape in the intervention group as compared to the SOC group, including random effects for each cohort, cluster (school), and individual, to account for repeated measures. To account for non-random attrition, we incorporated inverse probability of censoring weights to upweight observations corresponding to adolescents who had a high probability of attrition (Rosenman et al., 2020). As a form of sensitivity analysis, a generalized estimating equation (GEE) model with the same random effects was also run. In Rosenman et al. (2020)’s study, a simple protocol for adjudicating internally contradictory responses from a participant was proposed and evaluated; the current study takes any response suggesting a rape occurred as an indication that a rape did occur even if other responses were contradictory. The models were implemented using R packages lme4 (Bates et al., 2015) and GEE (Carey, 2019), and both analyzed only the 3263 adolescents appearing at both baseline and follow-up.

The population denominator for these analyses is 4121, slightly different than the 4125 reported in our baseline paper. Upon linking the baseline and follow-up results, we discovered four duplicated records at baseline, none of which reported rape and one of which reported sexual assault. These were removed for these analyses.

### Exploratory Analyses of Additional Girls’ Outcomes

We conducted exploratory analysis by subgroups, including (a) those who had previously reported experiencing rape and those who had not, (b) those with a history of violence in the home and without, (c) those living in each of the five communities in the study, and (d) economic status. For subgroup analyses, the Conditional Average Treatment Effect (CATE) estimates were GLMM coefficients from separate models for each subgroup. We examined changes in self-efficacy scores using a linear mixed model.

### Exploratory Analyses of Boys’ Outcomes

An exploratory analysis of the boys’ data assessed the intervention’s effect on (i) rates of sexual assault perpetration by the boys against any victim, and (ii) rates of sexual victimization of the boys by any perpetrator. The model accounted for time period, intervention status, and cohort using fixed effects, and accounted for repeated measures for each boy and school using a random effect.

## Results

### Demographics

At baseline, 82.9% of girls reported having both parents alive and 49.9% reported it would be difficult or very difficult to get 1000 Kenyan shillings for hospital or medicine (Table 1). The mean age was 11.7 years, with a range of 10–14 years. At the time of baseline data collection, the key variables of interest—rape in the last 12 months—was well-balanced, at 6.6% in the SOC group and 6.4% in the intervention group (standardized mean difference = 0.01).

**Table 1:** Baseline characteristics of girls

**Characteristic**	**Baseline (*n* = 3263)**	**Standard of care (*n* = 1541)**	**Treatment** **(*n* = 1722)**	**Standardized mean difference**
Parental status				
Both alive	2706 (82.9%)	1281 (83.1%)	1425 (82.8%)	0.06
Mother deceased	81 (2.5%)	45 (2.9%)	36 (2.1%)	
Father deceased	247 (7.6%)	116 (7.6%)	131 (7.6%)	
Orphaned	45 (1.4%)	18 (1.2%)	27 (1.6%)	
Do not know/not answered	184 (5.6%)	81 (5.3%)	103 (6.0%)	
Ability to get 1000 shillings for hospital or medicine				
Very difficult	623 (19.1%)	283 (18.4%)	340 (19.7%)	0.14
Difficult	1004 (30.8%)	452 (29.3%)	552 (32.1%)	
Easy	685 (21.0%)	311 (20.2%)	374 (21.7%)	
Very easy	187 (5.7%)	92 (6.0%)	95 (5.5%)	
Have medical insurance	756 (23.2%)	401 (26.0%)	355 (20.6%)	
Do not know/not answered	8 (0.2%)	2 (0.1%)	6 (0.3%)	
Have had a boyfriend				
Yes	654 (20.0%)	300 (19.5%)	354 (20.6%)	0.05
No	2526 (77.4%)	1205 (78.2%)	1321 (76.7%)	
Do not know/not answered	83 (2.6%)	36 (2.4%)	47 (2.7%)	
Raped in prior year				
Yes	212 (6.5%)	102 (6.6%)	110 (6.4%)	0.01
No	3051 (93.5%)	1439 (93.4%)	1612 (93.6%)	
Ever experienced rape				
Yes	301 (9.2%)	139 (9.0%)	162 (9.4%)	0.01
No	2962 (90.8%)	1402 (91.0%)	1560 (90.6%)	
Violence at home against mothers				
Yes	751 (23.0%)	352 (22.8%)	399 (23.2%)	0.01
No	2512 (77.0%)	1189 (77.2%)	1323 (76.8%)	
Prior No Means No Courses				
Yes	850 (26.3%)	381 (24.9%)	469 (27.4%)	0.13
No	2388 (73.7%)	1149 (75.1%)	1239 (72.5%)	

### Loss to Follow-up Among Girls

The 2-year retention rate was 79.2% among girls; no girls dropped out between recruitment and baseline measurements. Loss to follow-up was significantly correlated with baseline victimization status (Table 2). About 20% (*n* = 772) of adolescents who had never been raped at baseline were lost, compared to 27% (*n* = 72) who had been raped 1–4 times, and about 45% (14) who had been raped over five times at baseline (*p* < 0.001). Loss to follow-up was also significantly correlated with the trial arm (*p* < 0.05) with retention of 77.9% (1541/1979) in the SOC arm and 80.4% (1722/2142) in the treatment arm.

**Table 2:** Self-reported experiences of violence, by study arm

	**Baseline**		**Follow-up**	
**Characteristic**	**Standard of care (*n* = 1541)**	**Treatment (*n* = 1722)**	**Standard of care (*n* = 1541)**	**Treatment (*n* = 1722)**
Have had a boyfriend	300 (19.5%)	354 (20.6%)	429 (27.8%)	502 (29.2%)
Violence at home against mothers	352 (22.8%)	399 (23.2%)	231 (15.0%)	262 (15.2%)
Sexual assault (prior 12 months)	148 (9.6%)	175 (10.2%)	143 (9.3%)	167 (9.7%)
Rape (prior 12 months)	102 (6.6%)	110 (6.4%)	91 (5.9%)	103 (6.0%)
Sexual IPV*	48 (16.0%)	60 (16.9%)	61 (14.2%)	65 (12.9%)
Physical IPV	61 (20.3%)	73 (20.6%)	48 (11.2%)	69 (13.7%)
Emotional IPV	100 (33.3%)	108 (30.5%)	117 (27.3%)	121 (24.1%)

^*^IPV is reported with a denominator of the number of girls at baseline or endline, respectively, who reported having had a boyfriend in their lifetime

### Primary Analysis: Impact of Intervention

At follow-up, 5.9% (194/3263) of adolescent girls reported rape in the last 12 months, with 58.2% (113/194) reporting one rape and 6.2% (12/194) reporting five or more rapes. Among adolescents in intervention schools, 6.0% (103/1722) reported any rapes, versus 5.9% (91/1541) in SOC schools (Table 2).

Neither the GLMM nor GEE models resulted in a statistically significant difference between intervention and SOC groups. The GLMM estimated an odds ratio of 1.21 (*p* = 0.63, 95% CI (0.40, 5.21)) and the GEE estimated 1.05 (*p* = 0.75, 95% CI (0.65, 1.72)). These estimates are in the “increased rate of rape” direction. As some girls reported having been previously taught ESD skills (26.3% SOC, 27.4% treatment), we conducted a sensitivity analysis based on students’ self-reported prior exposure to ESD programs. This involved re-running our models excluding four schools where at least 70% of adolescents reported having the skills. No intervention effect was detected.

### Intimate Partner Violence

At follow-up, among girls reporting having ever had a boyfriend (28.5%, 931), 12.6% (117) reported physical IPV, 25.6% (238) reported emotional IPV, and 13.5% (126) reported sexual IPV in the previous 12 months (Table 2). There were no significant differences between study arms in reported rates of IPV at follow-up (Table 2).

### Secondary Analysis: Self-efficacy

While not significant, increases in social self-efficacy (OR: 1.08; 95% CI (0.95, 1.22), *p* = 0.22) and emotional self-efficacy (OR 1.07; 95% CI (0.89, 1.29), *p* = 0.49), as well as a decrease in academic self-efficacy (OR: 0.90; 95% CI (0.82, 1.00), *p* = 0.06), were observed (Table 3).

**Table 3:** GLMM and GEE results for secondary outcomes

**Outcome**	**Coefficient (GLMM)**	***p*-value (GLMM)**	**Coefficient (GEE)**	***p*-value (GEE)**
Self-efficacy (all)	1.01 (0.90, 1.14)	0.81	1.01 (0.90, 1.15)	0.84
Social self-efficacy	1.08 (0.95, 1.22)	0.21	1.08 (0.95, 1.22)	0.22
Emotional self-efficacy	1.07 (0.89, 1.29)	0.45	1.08 (0.91, 1.32)	0.44
Academic self-efficacy	0.90 (0.82, 1.00)	0.06	0.90 (0.83, 1.01)	0.06

### Subgroup Analysis: Violence at Home

Witnessing violence was common, with 15.1% (*n* = 493) of girls reporting at the endline data collection having seen their father or somebody else hitting their mother. This is lower than the rate reported at baseline, as previously published, of 23.0% (95% CI (21.6%, 24.1%) (Baiocchi et al., 2019). Between adolescents who witnessed such abuse and those who did not, rates of reported rape differed both at baseline (10.8% and 5.2%, respectively) and at follow-up (9.3% and 4.9%). Nonetheless, among those witnessing violence against their mothers, there were no statistically significant differences in rates of rape in the intervention group compared to the SOC (see Supplementary Information, Table S4).

### Subgroup Analysis: Re-victimization

It is possible that interventions for primary and secondary prevention of sexual assault may require different approaches. Therefore, we analyzed the effect of the intervention separately for adolescents who reported no rape at baseline versus those who reported at least one previous rape.

Among adolescents who reported never experiencing rape at baseline (*n* = 2,962), the rate of rape at follow-up for those receiving the SOC was 4.5% (*n* = 63) versus 4.6% (*n* = 72) for those receiving the intervention. Among adolescents who reported at least one prior experience of rape at baseline, the follow-up victimization rates were 20.1% (*n* = 28) for those in the SOC group, and 19.1% (*n* = 31) for those in the intervention group. As these data demonstrate, girls who had been raped at baseline were much more likely to experience a new rape during the follow-up. At follow-up, 42.9% of adolescents who reported being raped in the prior year reported that they had been raped more than once.

### Boys’ Sub-study

A total of 1104 boys completed the baseline survey and, of those, 832 completed the follow-up survey and were analyzed, for a 75.4% retention rate over 2 years. The mean age was 11.9 years, ranging from 10 to 14 years. Table S2 in the Supplementary Information describes the demographic characteristics of participants in the intervention and SOC groups at baseline, as well as the standardized mean difference for each covariate.

At baseline, 7.3% (*n* = 61/832, 95% CI (5.1%, 10.2%)) of adolescent boys reported experiencing rape, and 6.5% (*n* = 54/832, 95% CI (3.8%, 9.1%)) reported perpetrating rape, both in the last 12 months. We flag this rate as high and suggest interpretations in “Discussion.” Boys in the intervention group reported non-significantly higher rates of perpetration of rape at follow-up (5.7%, *n* = 25/436, 95% CI (2.9%, 8.7%)), compared to boys not receiving the intervention (3.8%, *n* = 15/396, 95% CI (1.6%, 6.6%), *p* = 0.14). Boys in the intervention group also reported higher rates of victimization at follow-up (5.7%, *n* = 25/436, 95% (CI 2.7%, 9.5%)), compared to boys not receiving the intervention (5.6%, *n* = 22/396, 95% CI (2.5%, 8.6%)), but again the intervention effect was not statistically significant (OR 6.93; 95% CI (0.50, 112.4), *p* = 0.06). We note that the differences between the groups are largely driven by differences in baseline rates, rather than follow-up differences; thus, caution should be taken in interpreting these results.

## Discussion

The high rate of sexual assault among young adolescent girls living in urban informal settlements represents a fundamental violation of human rights. These rates demonstrate a dire need for funders, policymakers, and the global health community to dedicate resources to sexual assault prevention, care, and treatment, and to include young adolescents in those services. We did not find evidence that the intervention studied, as implemented here, reduced sexual assault in the participant population.

Due to these unexpected findings, this study underwent two independent, external reviews performed by two separate teams: one from the South African Medical Research Council and a second from the London School of Hygiene and Tropical Medicine (LSHTM). Both teams reviewed our surveys, data, and code, interviewed study staff, and agreed that the study design, implementation, and analyses were sound. Both teams further concluded that the data collected does not provide evidence that the interventions reduced the rate of rape for girls, but both the primary funder (DFID) and the LSHTM team requested that methodological limitations be clearly outlined in this results paper.

Several factors may explain the differences between the findings here and in other studies of ESD. First, only three of the previous studies (Baiocchi et al., 2017; Decker et al., 2018; Senn et al., 2015) included randomization, and both in the African context were open cohorts without longitudinal follow-up. In open-cohort designs, even if the balance between the arms is achieved at baseline, differential changes in the composition may lead to incomparable groups at the follow-up assessment. Monitoring participants longitudinally allows the evaluation of non-random dropout rates and subgroup analyses. Here, dropout rates were significantly higher among adolescents who were experiencing the largest number of rapes at baseline. As efforts were made to find adolescents who transferred to other schools, it is likely that many who were lost had left school entirely. Therefore, it is likely that studies without longitudinal follow-up underweighted the impact of adolescents in high-risk situations. Previous studies could only have estimated an unbiased effect for the girls assuming there was no differential dropout between arms, but it would be impossible for those studies to estimate an unbiased effect if dropout were correlated with risk and heterogeneous response to the intervention (e.g., if girls frequently experiencing assaults were both non-responsive to the intervention and also more likely to drop out of the study). Average treatment effect estimates are only meaningful if they reflect the efficacy among the most vulnerable subpopulations.

Second, this is the first study of an ESD intervention in this setting using an independent data collection team. Previous studies in Nairobi and Malawi used the intervention trainers as data collectors. Demand effects, where research participants report what they think or know the investigators want to hear, are likely to be magnified when the same team implementing the intervention also collects the data (Higgins et al., 2008; McCambridge et al., 2014)). Thus, independent data collection teams are essential.

Third, much of the research showing the effectiveness of ESD has been in older populations, especially in universities, and, to a lesser degree, in secondary schools. We targeted young adolescents, 10–14 years of age, because sexual assault rates in secondary school have been reported as high as 25% annually in these communities (Sarnquist et al., 2014a, b), so it is essential to reach young people before they enter this especially high-risk time. However, adolescents in this younger age group may be less able to effectively deploy ESD strategies than older adolescents and young women, likely due to differences in physical, emotional, and cognitive development. While this may explain differences between this study and studies of older populations, it does not address the divergence from results in previous studies in this age group (Baiocchi et al., 2017; Jordan & Mossman, 2018). It seems likely that while ESD may be one approach to sexual assault prevention for this population, it should not be the only approach. This is consistent with many other sexual assault prevention programs and theories that have or recommend multiple components be employed to prevent sexual assault, and frequently include parents in this age group (DeGue et al., 2021; Heise, 1998; Jewkes et al., 2014; Kyegombe et al., 2014; Orchowski et al., 2018). Interventions that are more structural—built around organizational climate and leadership (Skopp et al., 2020), strictly enforced policies and procedures for interactions with at-risk adolescents (Cubellis, 2015), and environmental strategies (Taylor et al., 2013) —have been developed and may offer other pathways to reducing sexual assault.

The high prevalence of sexual assault among the young population in this study illustrates a dire need for effective interventions in this age group. Equally concerning, albeit consistent with other literature showing that prior rape is a key risk factor for future victimization (United Nations Children’s Fund, 2014), almost 20% of adolescent girls who had been raped at baseline experienced a new rape in the follow-up period. Furthermore, 42.9% of those reporting being raped in the prior year reported that it happened more than once. These data suggest myriad, severe risks and trauma to a fairly large number of girls, which will have negative implications across their lives. It is worth noting that prior ESD research has suggested reduced negative mental health outcomes, including self-blame, for young women who underwent ESD training but were still raped than those who had not undergone the training (Senn et al., 2017). Nonetheless, the high prevalence and incidence of rape in this young age group underscores the need for funders, policymakers, and practitioners to better address sexual assault prevention in this age group, tailored to both the general population and those who find themselves in situations of ongoing abuse.

Our findings also suggest high rates of sexual violence victimization (around 7%) among adolescent boys. This rate is higher than some, but not all, previous studies in boys and young men. There is a paucity of quality data on sexual assault of boys, and what exists likely suffers significantly from underreporting. Age is important: young adolescent boys, between 10 and 14 at baseline such as in our study, may be at similar risk of sexual assault as girls. Rates similar to those in our study have been reported. A South African study reported a 6.8% (101/1475 participants) “contact sexual abuse” rate among 14-year-old boys (Meinck et al., 2017). In the Pikine district in Senegal, reported rape rates for girls and boys 10–19 were not significantly different, at 3.8% and 2.8%, respectively (OR = 0.84, 0.38–1.84) (Anwar et al., 2020). A study in the USA found that in a large cohort of men, 6.7% reported having been raped in childhood (Dube et al., 2005). Some studies have also found high risk in young adult men; for example, a 2020 study at a US university reported a 13.7% rape rate for men in fraternities (Luetke et al., 2020). All these results, including ours, suggest that rape among adolescent males may be poorly characterized. Reliable measures of victimization and perpetration urgently need to be proposed and evaluated. Absent such measures, it is possible that male children and adolescents bear a larger burden of sexual victimization than is commonly acknowledged.

The findings of increased victimization and perpetration among boys are concerning but difficult to interpret, due to the wide confidence intervals and the fact that reliable data collection on sexual behavior from adolescent boys is challenging (Catania et al., 1990; Kelly et al., 2013; Langhaug et al., 2011; Palen et al., 2008; Phillips et al., 2010). Furthermore, neither the SOS program nor the study was designed specifically to affect or understand rape victimization or perpetration in boys. Given that this was one of the first deployments of this version of the boys’ intervention, caution needs to be used in interpreting this result. Nonetheless, the data presented suggest that the boys’ intervention should be both reviewed to ensure it incorporates the latest science on prevention for adolescent boys and studied independently in a trial designed for that purpose.

## Limitations

Several key limitations of this study include (i) the possibility of intervention diffusion into the community which could have biased the findings towards the null, (ii) the young age of participants, (iii) election violence which may have affected rates of sexual violence, (iv) the unexpected and difficult-to-explain finding that the reports of rape in the SOC group dropped over the study period, (v) response bias (e.g., “underreporting”) may have occurred, and (vi) the inability to use electronic data collection systems in this high-risk environment.

First, contamination was possible between intervention and SOC schools. Urban informal settlements are densely populated, and their populations are highly mobile, making it difficult to minimize diffusion of knowledge from the intervention group. Additionally, versions of the intervention have been taught in these communities since 2012, and thousands of adolescents have been trained; skills may have already diffused into the community. Moreover, local perpetrators may have learned the skills being taught and developed countermeasures. Testing in new settings would avoid this issue.

Second, knowledge about how ESD and other interventions affect and are understood by young adolescents, such as those included in this study, remains limited. It is possible that our results are affected by biases common to self-reported measures of sensitive topics—including response, social desirability, and recall biases.

Third, the study was likely affected by the violence surrounding Kenya’s 2017 national election. The resulting upheaval yielded many social and safety threats beyond the scope of this study to measure. The violence may have directly impacted our study outcome, but was unlikely to have impacted our study differentially by arm (McConnell, 2018).

Fourth, in the SOC group, there was an unexpected decline in the rate of reported rapes at follow-up compared to baseline, similar to the decrease in the intervention group. Previous studies suggest that sexual violence rates increase as adolescents age, although this effect has been described in secondary school rather than across upper primary years. We have not observed a temporal decrease in any prior studies that employed a similar intervention. Those other studies did not track individuals through time, so it is possible that a temporal effect was obscured by uncontrolled exit and entry of participants.

Finally, it is challenging to collect information on sexual violence, particularly in these settings and in this age group. Social desirability or discomfort with reporting may have led to biased responses. Due to logistical and financial constraints, we experienced a challenge in achieving the desired ratio of students to enumerators (i.e., 4:1) and ended up with a higher ratio (between 7.4:1 and 9.2:1), which may have increased discomfort in reporting on this quantitative, paper-based survey. In the baseline data, we also noted logical inconsistencies for some participants in their reports of victimization as well as sexual debut (Rosenman et al., 2020). Measurement error in the outcome measure can lower statistical power; though subsequent methodological work on this topic by our team suggests that this study may still have been well powered to detect an effect even in the presence of underreporting (Rosenman et al., 2023). There is a need for reliable self-reporting metrics of sensitive events for this age group in these settings, including cognitive behavioral evaluations of new metrics, and rates of sensitivity and specificity.

All of these limitations also apply to the boys’ exploratory study. For the boys, the reported rates of rape are quite high; as noted, there is existing literature demonstrating challenges with obtaining reliable information on these topics from adolescent boys.

## Conclusions

This is the largest and most rigorous evaluation of an ESD intervention with young adolescents in a low-income setting to date, with a cluster-randomized controlled trial design, large sample size, rigorous randomization, and longitudinal follow-up. In this age group and setting, we did not find evidence of the effectiveness of this intervention, although methodological and other limitations, as described above, must be considered in interpreting these results. Nonetheless, the alarmingly high rates of sexual assault among this young population delineates the dire need for increased funding and policy attention to implementing, and rigorously evaluating, sexual assault prevention interventions. It is possible that interventions that are more upstream of the adolescents themselves could be impactful in a way that the IMPower and SOS were not.

Sexual violence is deeply entrenched in many communities worldwide, including in complex environments such as informal settlements. Although the costs of sexual violence to both individuals and societies are extremely high, and the need for effective prevention strategies is urgent, this remains a relatively new field of empirical research. The results presented here underscore the need for careful evaluation of interventions, both as they scale up and are deployed in new populations, such as in this young age group and in a dense urban environment, in order to allocate resources most effectively.

### Supplementary Information

Below is the link to the electronic supplementary material.

Supplementary file1 (DOCX 88 KB)
