# Supplementary material for: Sexual Assault Among Young Adolescents in Informal Settlements in Nairobi, Kenya: Findings from the IMPower and SOS Cluster-Randomized Controlled Trial
Source: Prev Sci. 2023 Nov 15;25(4):578–89. doi: 10.1007/s11121-023-01595-1 (PMC11111490; doi:10.1007/s11121-023-01595-1)
Supplement: Supplementary file 1 — Supplementary file1 (DOCX 88 KB) [file 11121_2023_1595_MOESM1_ESM.docx]

**Title:** Appendices to Sexual assault among young adolescents in informal settlements in Nairobi, Kenya: Findings from the IMPower and SOS cluster-randomized controlled trial

**Authors:** Clea Sarnquist, DrPH^5*^, Rina Friedberg, PhD^2^, Evan Rosenman, PhD^3^, Mary Amuyunzu-Nyamongo, PhD^4^, Gavin Nyairo, and Michael Baiocchi, PhD^1,2^

1) Stanford University, Department of Epidemiology and Population Health

150 Governor’s Lane, Stanford, CA 94305-5405

2) LinkedIn Data Science and Applied Research

1000 West Maude Ave., Sunnyvale, CA 94085

3) Harvard University, Data Science Initiative

8 Story Street Suite 380, Cambridge, MA 02138

4) Africa Institute for Health and Development

7th Floor Suite B, Commodore, Wood Avenue/Kindaruma Road Junction, Box 45259, Nairobi, Kenya

5) Stanford University School of Medicine, Department of Pediatrics,

300 Pasteur Drive, Stanford CA 94305-5208

* Corresponding author

**Tables and figures**

**Table S1. Questions used to measure victimization among girls. If girls responded that an incident had occurred at least once in the prior year, then they were counted as having been raped in the prior year.**

| **Question Text** | **Possible Responses** |
| --- | --- |
| In the past 12 months, how many times has a current or a previous boyfriend ever physically forced you to have sex when you did not want to? | Never, Once, 2-3 times, 4+ times, N/A |
| In the past 12 months, how many times has your current or a previous boyfriend used threats or intimidation to get you to have sex when you did not want to? | Never, Once, 2-3 times, 4+ times, N/A |
| In the past 12 months, how many times has a man who is NOT your boyfriend forced or persuaded you to have sex against your will? | Never, Once, 2-3 times, 4+ times, N/A |
| In the past 12 months how many times has any man or boy who is NOT your boyfriend forced you to have sex against your will when you were too drunk or drugged to refuse? | Never, Once, 2-3 times, 4+ times, N/A |
| In the last 12 months how many times did two or more men or boys force you to have sex with them at the same time against your will? | Never, Once, 2-3 times, 4+ times, N/A |
| In the past 12 months how many times did two or more men or boys force you to have sex with them at the same time against your will when you were too drunk or drugged to refuse? | Never, Once, 2-3 times, 4+ times, N/A |
| In the past 12 months how many times was there an occasion when you agreed to have sex with one man or boy and one or more others who you had not agreed to have sex with forced you to have sex with them as well? | Never, Once, 2-3 times, 4+ times, N/A |

**Table S2. Baseline demographic characteristics (Boys)**

|  | **Baseline (n=832)** | **Standard of Care  (n = 396)** | **Treatment**  **(n = 436)** | **Standardized Mean Difference** |
| --- | --- | --- | --- | --- |
| Parental status (n = 158)* | | | | |
| Both alive | 142 (89·9%) | 60 (88·2%) | 82 (91·1%) | 0·36 |
| Mother deceased | 3 (1·9%) | 0 (0·0%) | 3 (3·3%) |  |
| Father deceased | 7 (4·4%) | 4 (5·9%) | 3 (3·3%) |  |
| Orphaned | 1 (0·6%) | 1 (1·5%) | 0 (0·0%) |  |
| Do not know | 5 (3·1%) | 3 (4·4%) | 2 (2·2%) |  |
| Ability to get 1000 shillings for hospital or medicine | | | | |
| Very difficult | 170 (20·4%) | 79 (19·9%) | 91 (20·9%) | 0·12 |
| Difficult | 270 (32·5%) | 131 (33·1%) | 139 (31·9%) |  |
| Easy | 183 (22·0%) | 93 (23·5%) | 90 (20·6%) |  |
| Very easy | 80 (9·6%) | 34 (8·6%) | 46 (10·6%) |  |
| Have medical insurance | 128 (15·4%) | 58 (14·6%) | 70 (16·1%) |  |
| Do not know/Not answered | 1 (0·1%) | 1 (0·3%) | 0 (0·0%) |  |
| Have had a girlfriend | | | | |
| Yes | 284 (34·1%) | 133 (33·6%) | 151 (34·6%) | 0·15 |
| No | 534 (64·2%) | 255 (64·4%) | 279 (64·0%) |  |
| Do not know/Not answered | 14 (1·7%) | 8 (2·1%) | 6 (1·4%) |  |
| Rape victim in prior year | | | | |
| Yes | 61 (7·3%) | 38 (9·6%) | 23 (5·3%) | 0·17 |
| No | 771 (92·7%) | 358 (90·4%) | 413 (94·7%) |  |
| Rape perpetrator in prior year | | | | |
| Yes | 54 (6·5%) | 28 (7·1%) | 26 (6·0%) | 0·05 |
| No | 778 (93·5%) | 368 (92·9%) | 410 (94·0%) |  |

* Parental status was asked on only a subset of surveys provided to the boys.

**Table S3. Self-reported violence victimization, by study arm and endline reporting status**

|  | Baseline (students surveyed at endline), n =3263 | | | | Baseline (all students), n =4121 | | | |
| --- | --- | --- | --- | --- | --- | --- | --- | --- |
|  | Standard of Care (n = 1541) | | Treatment (n = 1722) | | Standard of Care (n = 1979) | | Treatment (n = 2142) | |
|  | n | % | n | % | n | % | n | % |
| Have had a boyfriend | 300/1532 | 19·6% (15·8, 24·0) | 354/1706 | 20·8% (16·9, 24·6) | 409/1966 | 20·8% (17·1, 24·2) | 449/2124 | 21·1% (17·7, 24·6) |
| Violence at home against mothers | 352/1541 | 22·8% (19·2, 26·6) | 399/1722 | 23·2% (20·1, 26·9) | 450/1979 | 22·7% (19·5, 26·7) | 497/2142 | 23·2% (20·3, 25·9) |
| Sexual assault (12 months) | 148/1541 | 9·6% (7·5, 12·1) | 175/1722 | 10·2% (8·1, 12·3) | 210/1979 | 10·6% (8·4, 13·1) | 242/2142 | 11·3% (9·1, 13·5) |
| Rape (12 months) | 102/1541 | 6·6% (4·8, 8·8) | 110/1722 | 6·4% (4·8, 8·3) | 147/1979 | 7·4% (5·5, 9·4) | 151/21422 | 7·0% (5·4, 8·9) |
| Sexual IPV* | 49/300 | 16·3% (10·2, 24·3) | 62/354 | 17·5% (11·5, 24·7) | 63/409 | 15·4% (10·2, 21·4) | 80/449 | 17·8% (13·1, 23·2) |
| Physical IPV* | 65/300 | 21·7% (15·8, 28·6) | 73/354 | 20·6% (14·6, 27·1) | 91/409 | 22·2% (16·6, 28·8) | 95/449 | 21·2% (15·5, 27·4) |
| Emotional IPV* | 103/300 | 34·3% (26·6, 42·6) | 108/354 | 30·5% (23·1, 38·5) | 131/409 | 32·0% (25·1, 38·6) | 137/449 | 30·5% (24·0, 36·9) |

*IPV is measured only among students who reported having had a boyfriend.

**Table S4. Sub-group analysis: Rates of rape by community, economic status, violence against mothers**

|  | **Baseline** | | **Follow-up** | | **ATE estimate** |
| --- | --- | --- | --- | --- | --- |
| **Community** | **Standard of Care (n=1541)** | **Treatment (n=1722)** | **Standard of Care (n=1541)** | **Treatment (n=1722)** |  |
| Dandora (n = 719) | 16 (4·6%) | 25 (6·7%) | 18 (5·2%) | 25 (6·7%) | -0·20 (p=0·77) |
| Huruma (n = 572) | 10 (5·5%) | 25 (6·4%) | 14 (7·7%) | 27 (6·9%) | -0·68 (p=0·37) |
| Kibera (n = 499) | 20 (9·8%) | 19 (6·4%) | 13 (6·4%) | 17 (5·8%) | 1·14 (p=0·13) |
| Korogocho (n = 630) | 27 (8·3%) | 20 (6·6%) | 24 (7·3%) | 12 (4·0%) | -1·03 (p=0·15) |
| Mukuru (n = 843) | 20 (6·0%) | 21 (5·8%) | 22 (4·6%) | 22 (6·1%) | 0·92 (p=0·15) |
| **Economic status** |  |  |  |  |  |
| Very difficult  (n = 623) | 34 (12·0%) | 24 (7·1%) | 18 (6·4%) | 30 (8·8%) | 3·22 (p < 0·01) |
| Quite difficult  (n = 1004) | 26 (5·8%) | 39 (7·1%) | 32 (7·1%) | 36 (6·5%) | -0·71 (p = 0·17) |
| Easy  (n = 685) | 15 (4·8%) | 17 (4·5%) | 17 (5·5%) | 17 (4·5%) | -0·56 (p = 0·49) |
| Very easy  (n = 187) | 11 (12·0%) | 8 (8·4%) | 8 (8·7%) | 4 (4·2%) | -0·59 (p = 0·61) |
| Have medical insurance  (n = 756) | 16 (4·0%) | 22 (6·2%) | 16 (4·0%) | 16 (4·5%) | -0·76 (p = 0·22) |
| **Violence at home against mothers** |  |  |  |  |  |
| Exposed (n = 751) | 39 (11·1%) | 42 (10·5%) | 30 (8·5%) | 40 (10·0%) | 0·87 (p=0·10) |
| Not exposed (n = 2512) | 63 (5·3%) | 68 (5·1%) | 61 (5·1%) | 63 (4·8%) | -0·12 (p=0·73) |


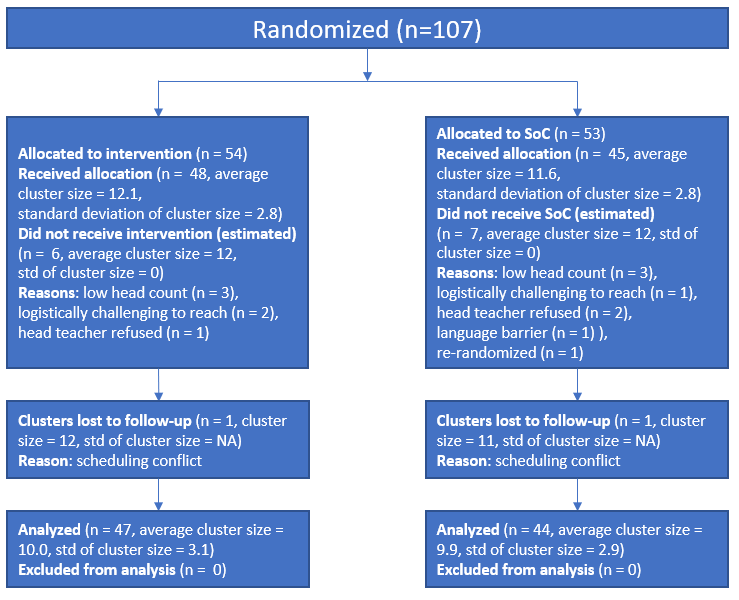


**Figure S1. Flow diagram for the exploratory boys study. School counts depart slightly from the girls study because one school was a girls-only school.**
